# 2-(2-Fur­yl)-1*H*-imidazo[4,5-*f*][1,10]phenanthroline-3,7-diium dichloride monohydrate

**DOI:** 10.1107/S1600536809001160

**Published:** 2009-01-17

**Authors:** Ming-Hua Chen, Yun-Qian Zhang, Qian-Jiang Zhu, Sai-Feng Xue, Zhu Tao

**Affiliations:** a Key Laboratory of Macrocyclic and Supramolecular Chemistry of Guizhou Province, Guizhou University, Guiyang 550025, People’s Republic of China; b Institute of Applied Chemistry, Guizhou University, Guiyang 550025, People’s Republic of China

## Abstract

The organic cation of the title salt, C_17_H_12_N_4_O^2+^·2Cl^−^·H_2_O, is nearly planar, the dihedral angle between two pyridine rings being 2.53 (16)° and that between the pyridinum and furan rings being 4.17 (19)°. Mol­ecules are linked *via* N—H⋯O, N—H⋯Cl and O—H⋯Cl hydrogen bonds, forming a three-dimensional framework and π–π stacking inter­actions help to stabilize the crystal structure [the imidazole–pyridine and imidazole–benzene centroid–centroid distances are 3.501 (3) and 3.674 (3) Å; respectively].

## Related literature

For general background, see: Zhao *et al.* (2004 ▶); Zheng *et al.* (2005 ▶).
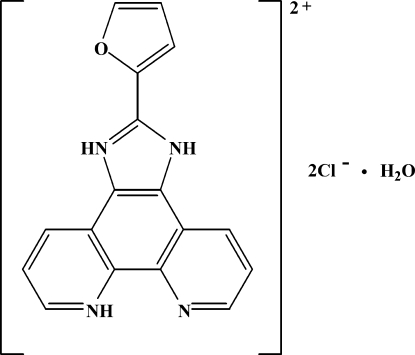


## Experimental

### 

#### Crystal data

C_17_H_12_N_4_O^2+^·2Cl^−^·H_2_O
*M* *_r_* = 377.22
Monoclinic, 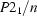

*a* = 4.768 (3) Å
*b* = 17.897 (10) Å
*c* = 19.241 (11) Å
β = 92.060 (8)°
*V* = 1641.0 (16) Å^3^
*Z* = 4
Mo *K*α radiation
μ = 0.42 mm^−1^
*T* = 293 (2) K
0.23 × 0.18 × 0.15 mm

#### Data collection

Bruker APEXII CCD area-detector diffractometer
Absorption correction: multi-scan (*SADABS*; Bruker, 2005 ▶) *T*_min_ = 0.911, *T*_max_ = 0.940
10782 measured reflections
2859 independent reflections
1886 reflections with *I* > 2σ(*I*)
*R*_int_ = 0.058

#### Refinement

*R*[*F* ^2^ > 2σ(*F* ^2^)] = 0.047
*wR*(*F* ^2^) = 0.115
*S* = 1.02
2859 reflections
226 parameters
H-atom parameters constrained
Δρ_max_ = 0.19 e Å^−3^
Δρ_min_ = −0.26 e Å^−3^

### 

Data collection: *APEX2* (Bruker, 2005 ▶); cell refinement: *SAINT* (Bruker, 2005 ▶); data reduction: *SAINT*; program(s) used to solve structure: *SHELXS97* (Sheldrick, 2008 ▶); program(s) used to refine structure: *SHELXL97* (Sheldrick, 2008 ▶); molecular graphics: *ORTEP-3 for Windows* (Farrugia, 1997 ▶); software used to prepare material for publication: *WinGX* (Farrugia, 1999 ▶).

## Supplementary Material

Crystal structure: contains datablocks global, I. DOI: 10.1107/S1600536809001160/at2707sup1.cif

Structure factors: contains datablocks I. DOI: 10.1107/S1600536809001160/at2707Isup2.hkl

Additional supplementary materials:  crystallographic information; 3D view; checkCIF report

## Figures and Tables

**Table 1:** Hydrogen-bond geometry (Å, °)

*D*—H⋯*A*	*D*—H	H⋯*A*	*D*⋯*A*	*D*—H⋯*A*
N1—H1*A*⋯O1*W*	0.86	1.76	2.618 (3)	177
N2—H2*A*⋯Cl1	0.86	2.12	2.981 (3)	179
N4—H4⋯Cl2^i^	0.86	2.33	3.103 (3)	150
O1*W*—H1*WA*⋯Cl2^ii^	1.02	2.00	3.011 (3)	172
O1*W*—H1*WB*⋯Cl2^iii^	0.93	2.19	3.112 (3)	173

Symmetry codes: (i) 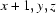; (ii) 
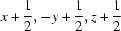
; (iii) 
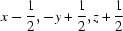
.

**Fig. 1.**
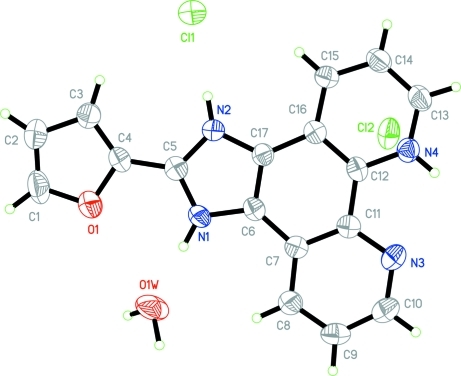
The molecular structure of (I) showing the atom-labelling scheme. Displacement ellipsoids are drawn at the 50% probability level.

## supplementary crystallographic information

### Comment

Recent year, we used different alkyl-substituted glycolurils as the building blocks to synthesize the partially alkyl substituted cucurbit[*n*]urils (Zhao *et al.*, 2004; Zheng *et al.*, 2005). In this work, we further report the crystal structure of a phenanthroline-substituted semi-glycoluril.

The molecular structure of the title compound (I), consists of organic cations, two Cl^-^ anions and one water molecule (Fig. 1). The organic cation is nearly planar, the dihedral angle between two pyridine rings is 2.53 (16) ° and that one between N4-pyridine ring and furan ring is 4.17 (19)°. Molecules are linked *via* N1—H1A···O1W, N2—H2A···Cl1, N4—H4···Cl2, O1W—H1WA···Cl2 and O1W—H1WB···Cl2 hydrogen bonds (Table 1) forming a three-dimensional framework. In additional, the π···π stacking interaction occurs between adjacent organic cations, the centroid-to-centroid distance of *Cg*2 to *Cg*3^iv^ is 3.501 (3)Å and *Cg*2 to *Cg*5^iv^ is 3.674 (3)Å [*Cg*2, *Cg*3 and *Cg*5 is the centroid of the N1-imidazole ring, N3-pyridine ring and C6-benzene ring, respectively. Symmerty codes: (iv) -1 + *x*, *y*, *z*].

### Experimental

1,10-Phenanthroline-5,6-dione (1.0 g, 4.75 mmol) and ammonium acetate (3.9 g, 5 mmol) are dissolved in acetic acid glacial (60 mL) at 333 K, after cooling at room temperature, we can get lots of yellow deposits by the neutrlizing with ammonia to pH = 8–9 after 4 h of mixing with furaldhyde (0.8 mL) at 353 K. After the recryslalization in ethanol, the yellow product was dissolved in the dilute hydrochloric acid and red-brown crystals were obtained

after ten days.

### Refinement

Water H atoms were located in a difference Fourier synthesis and refined riding in their as-found positions relative to O atoms with *U*_iso_(H) =1.2*U*_eq_(O). All other H atoms were placed in calculated positions and refined as riding, with C—H = 0.93–0.97 Å, N—H = 0.86 Å, and with *U*_iso_(H) = 1.2 *U*_eq_ (C, N).

### Crystal data

**Table:**

C_17_H_12_N_4_O^2+^·2Cl^−^·H_2_O	*F*(000) = 776
*M**_r_* = 377.22	*D*_x_ = 1.527 Mg m^−^^3^
Monoclinic, *P*2_1_/*n*	Mo *K*α radiation, λ = 0.71073 Å
Hall symbol: -P 2yn	Cell parameters from 2859 reflections
*a* = 4.768 (3) Å	θ = 1.6–25.0°
*b* = 17.897 (10) Å	µ = 0.42 mm^−^^1^
*c* = 19.241 (11) Å	*T* = 293 K
β = 92.060 (8)°	Prism, red-brown
*V* = 1641.0 (16) Å^3^	0.23 × 0.18 × 0.15 mm
*Z* = 4	

### Data collection

**Table:**

Bruker APEXII CCD area-detector diffractometer	2859 independent reflections
Radiation source: fine-focus sealed tube	1886 reflections with *I* > 2σ(*I*)
graphite	*R*_int_ = 0.058
φ and ω scans	θ_max_ = 25.0°, θ_min_ = 1.6°
Absorption correction: multi-scan (*SADABS*; Bruker, 2005)	*h* = −5→5
*T*_min_ = 0.911, *T*_max_ = 0.940	*k* = −20→21
10782 measured reflections	*l* = −22→22

### Refinement

**Table:**

Refinement on *F*^2^	Primary atom site location: structure-invariant direct methods
Least-squares matrix: full	Secondary atom site location: difference Fourier map
*R*[*F*^2^ > 2σ(*F*^2^)] = 0.047	Hydrogen site location: inferred from neighbouring sites
*wR*(*F*^2^) = 0.115	H-atom parameters constrained
*S* = 1.02	*w* = 1/[σ^2^(*F*_o_^2^) + (0.0514*P*)^2^] where *P* = (*F*_o_^2^ + 2*F*_c_^2^)/3
2859 reflections	(Δ/σ)_max_ < 0.001
226 parameters	Δρ_max_ = 0.19 e Å^−^^3^
0 restraints	Δρ_min_ = −0.26 e Å^−^^3^

### Special details

**Table:**

Geometry. All e.s.d.'s (except the e.s.d. in the dihedral angle between two l.s. planes) are estimated using the full covariance matrix. The cell e.s.d.'s are taken into account individually in the estimation of e.s.d.'s in distances, angles and torsion angles; correlations between e.s.d.'s in cell parameters are only used when they are defined by crystal symmetry. An approximate (isotropic) treatment of cell e.s.d.'s is used for estimating e.s.d.'s involving l.s. planes.
Refinement. Refinement of *F*^2^ against ALL reflections. The weighted *R*-factor *wR* and goodness of fit *S* are based on *F*^2^, conventional *R*-factors *R* are based on *F*, with *F* set to zero for negative *F*^2^. The threshold expression of *F*^2^ > σ(*F*^2^) is used only for calculating *R*-factors(gt) *etc*. and is not relevant to the choice of reflections for refinement. *R*-factors based on *F*^2^ are statistically about twice as large as those based on *F*, and *R*- factors based on ALL data will be even larger.

### Fractional atomic coordinates and isotropic or equivalent isotropic displacement parameters (Å^2^)

**Table:**

	*x*	*y*	*z*	*U*_iso_*/*U*_eq_	
C1	0.2549 (7)	0.5257 (2)	0.91899 (18)	0.0595 (10)	
H1	0.1981	0.5270	0.9647	0.071*	
C2	0.1543 (7)	0.57043 (18)	0.86872 (17)	0.0491 (9)	
H2	0.0184	0.6073	0.8728	0.059*	
C3	0.2960 (6)	0.55060 (16)	0.80743 (16)	0.0439 (8)	
H3	0.2711	0.5718	0.7635	0.053*	
C4	0.4742 (6)	0.49469 (16)	0.82609 (15)	0.0366 (7)	
C5	0.6721 (6)	0.45050 (15)	0.78964 (15)	0.0356 (7)	
C6	0.9978 (6)	0.36694 (15)	0.76729 (14)	0.0334 (7)	
C7	1.2049 (6)	0.30919 (15)	0.77145 (14)	0.0331 (7)	
C8	1.2878 (6)	0.26901 (16)	0.83160 (16)	0.0405 (8)	
H8	1.2062	0.2786	0.8739	0.049*	
C9	1.4924 (6)	0.21529 (17)	0.82645 (16)	0.0465 (8)	
H9	1.5506	0.1877	0.8653	0.056*	
C10	1.6108 (6)	0.20276 (16)	0.76279 (17)	0.0453 (8)	
H10	1.7500	0.1665	0.7607	0.054*	
C11	1.3351 (6)	0.29168 (15)	0.70909 (14)	0.0328 (7)	
C12	1.2531 (6)	0.33026 (15)	0.64536 (14)	0.0320 (7)	
C13	1.3161 (6)	0.34323 (16)	0.52471 (15)	0.0417 (8)	
H13	1.4109	0.3287	0.4855	0.050*	
C14	1.1068 (6)	0.39700 (16)	0.51891 (15)	0.0420 (8)	
H14	1.0577	0.4177	0.4758	0.050*	
C15	0.9734 (6)	0.41932 (15)	0.57711 (14)	0.0376 (7)	
H15	0.8349	0.4559	0.5741	0.045*	
C16	1.0477 (6)	0.38639 (15)	0.64198 (14)	0.0322 (7)	
C17	0.9259 (6)	0.40383 (15)	0.70643 (14)	0.0319 (7)	
N3	1.5391 (5)	0.23910 (13)	0.70414 (13)	0.0412 (6)	
N4	1.3821 (5)	0.31224 (12)	0.58612 (12)	0.0359 (6)	
H4	1.5130	0.2792	0.5882	0.043*	
N1	0.8358 (5)	0.39716 (13)	0.81854 (11)	0.0348 (6)	
H1A	0.8394	0.3840	0.8615	0.042*	
N2	0.7228 (5)	0.45605 (12)	0.72179 (12)	0.0345 (6)	
H2A	0.6432	0.4867	0.6928	0.041*	
O1	0.4528 (5)	0.47741 (12)	0.89489 (11)	0.0550 (6)	
Cl1	0.44997 (17)	0.56432 (4)	0.62293 (4)	0.0514 (3)	
Cl2	0.83425 (17)	0.20341 (5)	0.53339 (5)	0.0579 (3)	
O1W	0.8356 (5)	0.35229 (14)	0.94806 (11)	0.0703 (8)	
H1WA	0.9993	0.3370	0.9803	0.084*	
H1WB	0.6840	0.3400	0.9751	0.084*	

### Atomic displacement parameters (Å^2^)

**Table:**

	*U*^11^	*U*^22^	*U*^33^	*U*^12^	*U*^13^	*U*^23^
C1	0.053 (2)	0.077 (3)	0.049 (2)	0.008 (2)	0.0175 (18)	−0.016 (2)
C2	0.047 (2)	0.046 (2)	0.055 (2)	0.0040 (16)	0.0111 (17)	−0.0081 (17)
C3	0.048 (2)	0.0404 (19)	0.044 (2)	0.0041 (16)	0.0072 (16)	0.0008 (15)
C4	0.0363 (17)	0.0435 (18)	0.0304 (17)	−0.0035 (15)	0.0052 (14)	−0.0034 (14)
C5	0.0371 (18)	0.0338 (17)	0.0360 (19)	−0.0036 (14)	0.0023 (14)	−0.0014 (13)
C6	0.0325 (16)	0.0372 (17)	0.0307 (17)	−0.0030 (14)	0.0030 (13)	−0.0017 (13)
C7	0.0321 (16)	0.0339 (17)	0.0331 (18)	−0.0038 (13)	0.0004 (13)	0.0010 (13)
C8	0.0395 (18)	0.0436 (19)	0.0387 (18)	0.0014 (15)	0.0033 (14)	0.0035 (15)
C9	0.051 (2)	0.046 (2)	0.042 (2)	0.0001 (16)	−0.0055 (16)	0.0126 (15)
C10	0.0442 (19)	0.0371 (19)	0.054 (2)	0.0061 (15)	−0.0062 (17)	−0.0002 (16)
C11	0.0309 (16)	0.0328 (17)	0.0344 (18)	−0.0052 (13)	−0.0028 (13)	−0.0023 (13)
C12	0.0302 (16)	0.0345 (17)	0.0314 (17)	−0.0060 (13)	0.0043 (13)	−0.0048 (13)
C13	0.049 (2)	0.0446 (19)	0.0318 (18)	−0.0005 (16)	0.0065 (15)	−0.0037 (14)
C14	0.053 (2)	0.0402 (19)	0.0327 (18)	−0.0001 (16)	0.0047 (15)	0.0011 (14)
C15	0.0422 (18)	0.0368 (18)	0.0339 (18)	0.0011 (14)	0.0025 (14)	0.0043 (14)
C16	0.0331 (16)	0.0308 (16)	0.0329 (17)	−0.0045 (13)	0.0019 (13)	−0.0018 (13)
C17	0.0332 (16)	0.0310 (16)	0.0314 (17)	−0.0010 (13)	0.0010 (13)	−0.0022 (13)
N3	0.0389 (15)	0.0349 (15)	0.0497 (17)	0.0050 (12)	−0.0006 (12)	−0.0030 (12)
N4	0.0351 (14)	0.0377 (15)	0.0349 (15)	0.0019 (11)	0.0009 (11)	−0.0045 (11)
N1	0.0371 (14)	0.0396 (15)	0.0281 (14)	−0.0006 (12)	0.0062 (11)	0.0040 (11)
N2	0.0364 (14)	0.0356 (14)	0.0315 (15)	0.0042 (11)	0.0023 (11)	0.0033 (11)
O1	0.0560 (15)	0.0699 (16)	0.0400 (14)	0.0159 (12)	0.0122 (11)	0.0021 (11)
Cl1	0.0573 (5)	0.0518 (5)	0.0452 (5)	0.0106 (4)	0.0014 (4)	0.0082 (4)
Cl2	0.0407 (5)	0.0682 (6)	0.0651 (6)	0.0031 (4)	0.0057 (4)	−0.0194 (5)
O1W	0.0487 (15)	0.120 (2)	0.0432 (14)	0.0106 (14)	0.0133 (11)	0.0309 (14)

### Geometric parameters (Å, °)

**Table:**

C1—C2	1.332 (4)	C10—N3	1.336 (4)
C1—O1	1.373 (4)	C10—H10	0.9300
C1—H1	0.9300	C11—N3	1.359 (3)
C2—C3	1.425 (4)	C11—C12	1.449 (4)
C2—H2	0.9300	C12—N4	1.353 (3)
C3—C4	1.353 (4)	C12—C16	1.403 (4)
C3—H3	0.9300	C13—N4	1.333 (3)
C4—O1	1.367 (3)	C13—C14	1.388 (4)
C4—C5	1.434 (4)	C13—H13	0.9300
C5—N2	1.340 (3)	C14—C15	1.367 (4)
C5—N1	1.341 (3)	C14—H14	0.9300
C6—C17	1.377 (4)	C15—C16	1.414 (4)
C6—N1	1.384 (3)	C15—H15	0.9300
C6—C7	1.429 (4)	C16—C17	1.423 (4)
C7—C11	1.406 (4)	C17—N2	1.385 (3)
C7—C8	1.407 (4)	N4—H4	0.8600
C8—C9	1.376 (4)	N1—H1A	0.8600
C8—H8	0.9300	N2—H2A	0.8600
C9—C10	1.385 (4)	O1W—H1WA	1.0173
C9—H9	0.9300	O1W—H1WB	0.9323
			
C2—C1—O1	111.5 (3)	C7—C11—C12	120.1 (3)
C2—C1—H1	124.2	N4—C12—C16	118.0 (3)
O1—C1—H1	124.2	N4—C12—C11	118.8 (3)
C1—C2—C3	106.4 (3)	C16—C12—C11	123.2 (3)
C1—C2—H2	126.8	N4—C13—C14	120.4 (3)
C3—C2—H2	126.8	N4—C13—H13	119.8
C4—C3—C2	106.1 (3)	C14—C13—H13	119.8
C4—C3—H3	127.0	C15—C14—C13	119.4 (3)
C2—C3—H3	127.0	C15—C14—H14	120.3
C3—C4—O1	110.9 (3)	C13—C14—H14	120.3
C3—C4—C5	134.1 (3)	C14—C15—C16	119.5 (3)
O1—C4—C5	115.0 (3)	C14—C15—H15	120.3
N2—C5—N1	109.5 (3)	C16—C15—H15	120.3
N2—C5—C4	125.5 (3)	C12—C16—C15	119.5 (3)
N1—C5—C4	125.0 (3)	C12—C16—C17	115.0 (2)
C17—C6—N1	106.9 (2)	C15—C16—C17	125.5 (3)
C17—C6—C7	123.0 (3)	C6—C17—N2	107.3 (2)
N1—C6—C7	130.1 (3)	C6—C17—C16	122.6 (3)
C11—C7—C8	117.9 (3)	N2—C17—C16	130.1 (2)
C11—C7—C6	116.0 (2)	C10—N3—C11	116.1 (3)
C8—C7—C6	126.1 (3)	C13—N4—C12	123.2 (3)
C9—C8—C7	118.5 (3)	C13—N4—H4	118.4
C9—C8—H8	120.8	C12—N4—H4	118.4
C7—C8—H8	120.8	C5—N1—C6	108.3 (2)
C8—C9—C10	119.3 (3)	C5—N1—H1A	125.8
C8—C9—H9	120.3	C6—N1—H1A	125.8
C10—C9—H9	120.3	C5—N2—C17	108.1 (2)
N3—C10—C9	124.6 (3)	C5—N2—H2A	126.0
N3—C10—H10	117.7	C17—N2—H2A	126.0
C9—C10—H10	117.7	C4—O1—C1	105.1 (2)
N3—C11—C7	123.6 (3)	H1WA—O1W—H1WB	100.9
N3—C11—C12	116.3 (3)		
			
O1—C1—C2—C3	−0.3 (4)	C11—C12—C16—C17	0.8 (4)
C1—C2—C3—C4	−0.2 (4)	C14—C15—C16—C12	−1.3 (4)
C2—C3—C4—O1	0.5 (3)	C14—C15—C16—C17	−179.7 (3)
C2—C3—C4—C5	179.6 (3)	N1—C6—C17—N2	0.3 (3)
C3—C4—C5—N2	−0.9 (5)	C7—C6—C17—N2	−179.4 (2)
O1—C4—C5—N2	178.1 (3)	N1—C6—C17—C16	−178.8 (2)
C3—C4—C5—N1	178.9 (3)	C7—C6—C17—C16	1.4 (4)
O1—C4—C5—N1	−2.0 (4)	C12—C16—C17—C6	−1.9 (4)
C17—C6—C7—C11	0.2 (4)	C15—C16—C17—C6	176.6 (3)
N1—C6—C7—C11	−179.5 (3)	C12—C16—C17—N2	179.2 (3)
C17—C6—C7—C8	−179.7 (3)	C15—C16—C17—N2	−2.3 (5)
N1—C6—C7—C8	0.6 (5)	C9—C10—N3—C11	0.0 (4)
C11—C7—C8—C9	0.4 (4)	C7—C11—N3—C10	0.9 (4)
C6—C7—C8—C9	−179.6 (3)	C12—C11—N3—C10	−178.9 (2)
C7—C8—C9—C10	0.4 (4)	C14—C13—N4—C12	0.2 (4)
C8—C9—C10—N3	−0.6 (5)	C16—C12—N4—C13	−2.5 (4)
C8—C7—C11—N3	−1.1 (4)	C11—C12—N4—C13	178.2 (2)
C6—C7—C11—N3	179.0 (2)	N2—C5—N1—C6	−0.2 (3)
C8—C7—C11—C12	178.7 (2)	C4—C5—N1—C6	179.9 (3)
C6—C7—C11—C12	−1.3 (4)	C17—C6—N1—C5	−0.1 (3)
N3—C11—C12—N4	−0.2 (4)	C7—C6—N1—C5	179.6 (3)
C7—C11—C12—N4	180.0 (2)	N1—C5—N2—C17	0.4 (3)
N3—C11—C12—C16	−179.4 (2)	C4—C5—N2—C17	−179.7 (3)
C7—C11—C12—C16	0.8 (4)	C6—C17—N2—C5	−0.4 (3)
N4—C13—C14—C15	1.6 (4)	C16—C17—N2—C5	178.6 (3)
C13—C14—C15—C16	−1.0 (4)	C3—C4—O1—C1	−0.7 (3)
N4—C12—C16—C15	3.0 (4)	C5—C4—O1—C1	−180.0 (3)
C11—C12—C16—C15	−177.8 (2)	C2—C1—O1—C4	0.6 (4)
N4—C12—C16—C17	−178.5 (2)		

### Hydrogen-bond geometry (Å, °)

**Table:**

*D*—H···*A*	*D*—H	H···*A*	*D*···*A*	*D*—H···*A*
N1—H1A···O1W	0.86	1.76	2.618 (3)	177
N2—H2A···Cl1	0.86	2.12	2.981 (3)	179
N4—H4···Cl2^i^	0.86	2.33	3.103 (3)	150
O1W—H1WA···Cl2^ii^	1.02	2.00	3.011 (3)	172
O1W—H1WB···Cl2^iii^	0.93	2.19	3.112 (3)	173

Symmetry codes: (i) *x*+1, *y*, *z*; (ii) *x*+1/2, −*y*+1/2, *z*+1/2; (iii) *x*−1/2, −*y*+1/2, *z*+1/2.
